# Supplementary material for: Implications of being born late in the active season for growth, fattening, torpor use, winter survival and fecundity
Source: eLife. 2018 Feb 20;7:e31225. doi: 10.7554/eLife.31225 (PMC5819945; doi:10.7554/eLife.31225)
Supplement: Supplementary file 1. — Body mass was also assessed as an explanatory variable in the models. p-Values shown in bold correspond to statistically significant and interpretable values. [file elife-31225-supp1.docx]

**Table S1.** Parameters of linear models for the effects of group and diet on total energy expenditure and water turnover of juvenile garden dormice. Body mass was also assessed as an explanatory variable in the models. p-values shown in bold correspond to statistically significant and interpretable values.

| Response variable | Term | Estimate ± SD | t-value | p-value |
| --- | --- | --- | --- | --- |
|  |  |  |  |  |
| Total energy expenditure (KJ day^-1^) | Group | -0.19 ± 6.47 | -0.03 | 0.98 |
|  | Diet  Body Mass | -12.43 ± 4.86  0.28 ± 0.19 | -2.56  1.47 | < 0.05  0.16 |
|  | Group x Diet | 34.64 ± 7.02 | 4.93 | **< 0.001** |
|  |  |  |  |  |
| Water turnover (g day^-1^) | Group | -3.75 ± 1.50 | -2.51 | < 0.05 |
|  | Diet  Body Mass | -3.06 ± 1.12  0.01 ± 0.04 | -2.73  0.18 | < 0.05  0.86 |
|  | Group x Diet | 7.74 ± 1.62 | 4.76 | **< 0.001** |
|  |  |  |  |  |
