# Supplementary material for: Implications of being born late in the active season for growth, fattening, torpor use, winter survival and fecundity
Source: eLife. 2018 Feb 20;7:e31225. doi: 10.7554/eLife.31225 (PMC5819945; doi:10.7554/eLife.31225)
Supplement: Supplementary file 2. — Metabolic rate is expressed in ml O2 h−1 g−1. Body mass and animal ID were included as random effects in all models. p-Values shown in bold correspond to statistically significant and interpretable values. [file elife-31225-supp2.docx]

**Table S2.** Means ± standard errors and parameters of analyses of variance for the effects of group (early-born ‘EB’ *vs.* late-born ‘LB’), diet (*ad-libitum* ‘AL’ *vs.* intermittently fasted ‘IF’) and time (‘start of growth’ *vs.* ‘body mass plateau’) on average daily metabolic rate (‘ADMR’) of garden dormice, metabolic rate during night (‘MR-night’) and metabolic rate during day (‘MR-day’). Metabolic rate is expressed in ml O_2_ h^-1^ g^-1^. Body mass and animal ID were included as random effects in all models. p-values shown in bold correspond to statistically significant and interpretable values.

|  |
| --- |
| \|  \| \| --- \|  \| **Variable** \| **Time point** \| **EB-AL** \| **EB-IF** \| **LB-AL** \| **LB-IF** \| **ANOVA** \| \| \| \| \| \| \| --- \| --- \| --- \| --- \| --- \| --- \| --- \| --- \| --- \| --- \| --- \| --- \| \| **Group** \| \| **Diet** \| \| **Time** \| \| \| **χ ^2^** \| **p-value** \| **χ ^2^** \| **p-value** \| **χ ^2^** \| **p-value** \| \| **ADMR** \| **Start of growth**  **Body mass plateau** \| 3.0 ± 0.1  2.4 ± 0.2 \| 2.8 ± 0.1  2.2 ± 0.1 \| 3.5 ± 0.4  2.2 ± 0.2 \| 2.9 ± 0.2  2.3 ± 0.2 \| 1.23 \| 0.27 \| 2.19 \| 0.14 \| 23.42 \| **< 0.001** \| \| **MR-night** \| **Start of growth**  **Body mass plateau** \| 3.8 ± 0.1  2.6 ± 0.2 \| 3.7 ± 0.2  2.7 ± 0.1 \| 3.7 ± 0.4  2.4 ± 0.3 \| 3.4 ± 0.2  2.8 ± 0.1 \| 5.27 \| **< 0.05** \| 0.09 \| 0.76 \| 23.98 \| **< 0.001** \| \| **MR-day** \| **Start of growth**  **Body mass plateau** \| 2.6 ± 0.1  2.1 ± 0.2 \| 2.3 ± 0.1  1.8 ± 0.1 \| 3.3 ± 0.5  1.9 ± 0.2 \| 2.4 ± 0.3  1.8 ± 0.3 \| 0.88 \| 0.35 \| 5.80 \| **< 0.05** \| 11.50 \| **< 0.001** \| |
