# Supplementary material for: Implications of being born late in the active season for growth, fattening, torpor use, winter survival and fecundity
Source: eLife. 2018 Feb 20;7:e31225. doi: 10.7554/eLife.31225 (PMC5819945; doi:10.7554/eLife.31225)
Supplement: Supplementary file 3. [file elife-31225-supp3.docx]

**Table S3.** Means and standard errors for the time (‘starting time’) and rate (‘slope’) of increase in torpor frequency, mean and total torpor duration of early-born (‘EB’) and late-born (‘LB’) juvenile garden dormice, either fed *ad libitum* (‘AL’) or intermittently fasted (‘IF’).

| Variable | Group | Parameters  Starting time Slope  (weeks) | |
| --- | --- | --- | --- |
|  | |  |  |
|  | |  |  |
| Torpor frequency  (bouts week^-1^) | EB-AL | 8.50 ± 0.56 | 1.29 ± 0.21 |
|  | EB-IF | 6.50 ± 0.62 | 0.98 ± 0.17 |
|  | LB-AL | 4.17 ± 0.17 | 0.83 ± 0.17 |
|  | LB-IF | 3.67 ± 0.33 | 1.54 ± 0.22 |
|  |  |  |  |
| Mean torpor duration  (hours bout^-1^ week^-1^) | EB-AL | 8.50 ± 0.56 | 2.48 ± 0.67 |
|  | EB-IF | 6.50 ± 0.62 | 1.86 ± 0.47 |
|  | LB-AL | 4.17 ± 0.17 | 8.88 ± 1.19 |
|  | LB-IF | 3.00 ± 0.37 | 2.30 ± 0.24 |
|  |  |  |  |
| Total torpor duration  (hours week^-1^) | EB-AL | 8.50 ± 0.56 | 8.45 ± 1.84 |
|  | EB-IF | 6.50 ± 0.62 | 10.10 ± 1.78 |
|  | LB-AL | 4.17 ± 0.17 | 28.28 ± 1.71 |
|  | LB-IF | 3.00 ± 0.37 | 19.76 ± 0.22 |
