# Supplementary material for: Implications of being born late in the active season for growth, fattening, torpor use, winter survival and fecundity
Source: eLife. 2018 Feb 20;7:e31225. doi: 10.7554/eLife.31225 (PMC5819945; doi:10.7554/eLife.31225)
Supplement: Supplementary file 4. — Hibernation duration was also assed as an explanatory variable in the model for arousal frequency. p-Values shown in italic correspond to statistically significant and interpretable values. [file elife-31225-supp4.docx]

**Table S4.** Parameters of linear models for the effects of group and diet on the hibernation duration, arousal frequency, mean torpor and arousal durations and minimal body temperature (T_b_) during winter. Hibernation duration was also assed as an explanatory variable in the model for arousal frequency. p-values shown in italic correspond to statistically significant and interpretable values.

| Response variable | Term | Estimate  ± SD | Statistical value | p-value |
| --- | --- | --- | --- | --- |
|  |  |  |  |  |
| Hibernation duration | Group | -920.11 ± 12.83 | -71.72 | **< 0.001** |
|  | Diet | -15.38 ± 12.83 | -1.20 | 0.249 |
|  |  |  |  |  |
| Arousal frequency | Group | 36.19 ± 18.08 | 2.00 | 0.065 |
|  | Diet  Hibernation Duration | 0.21 ± 1.02  0.04 ± 0.02 | 0.21  2.43 | 0.839  **< 0.05** |
| Mean torpor duration | Group | 14.83 ± 12.00 | 1.24 | 0.235 |
|  | Diet | -0.13 ± 12.00 | -0.01 | 0.992 |
| Mean arousal duration | Group | -0.29 ± 0.35 | -0.82 | 0.423 |
|  | Diet | 0.18 ± 0.35 | 0.50 | 0.622 |
|  |  |  |  |  |
| Minimal T_b_ | Group | -0.01 ± 0.26 | -0.05 | 0.962 |
|  | Diet | 0.14 ± 0.26 | 0.52 | 0.613 |
|  |  |  |  |  |
|  |  |  |  |  |
