# Supplementary material for: Implications of being born late in the active season for growth, fattening, torpor use, winter survival and fecundity
Source: eLife. 2018 Feb 20;7:e31225. doi: 10.7554/eLife.31225 (PMC5819945; doi:10.7554/eLife.31225)
Supplement: Supplementary file 5. [file elife-31225-supp5.docx]

**Table S5.** Means and standard deviations for the hibernation duration, arousal frequency, mean torpor and arousal durations and minimal body temperature (T_b_) of juveniles during winter, according to their time of birth (early-born ‘EB’, late-born ‘LB’) and feeding treatment (*ad libitum ‘*AL’ and intermittently fasted ‘IF’).

| Variable | Group | Mean ± SD |
| --- | --- | --- |
|  |  |  |
|  |  |  |
| Hibernation duration (h) | EB | 3908.11 ± 32.56 |
|  | LB  AL  IF | 2986.07 ± 22.15  3453.82 ± 496.02  3323.42 ± 462.96 |
|  |  |  |
| Arousal frequency | EB  LB  AL  IF | 19.4 ± 2.4  13.8 ± 2.1  16.8 ± 4.4  15.6 ± 2.3 |
|  |  |  |
| Mean torpor duration (h) | EB  LB  AL  IF | 195.09 ± 21.40  209.91 ± 26.33  202.56 ± 30.32  204.28 ± 17.45 |
|  |  |  |
| Mean arousal duration (h) | EB  LB  AL  IF | 5.54 ± 0.96  5.27 ± 0.45  5.32 ± 0.77  5.46 ± 0.69 |
|  |  |  |
| Minimal T_b_ (°C) | EB  LB  AL  IF | 6.04 ± 0.77  6.04 ± 0.24  5.98 ± 0.53  6.12 ± 0.54 |
|  |  |  |
|  |  |  |
|  |  |  |
