# Supplementary material for: Implications of being born late in the active season for growth, fattening, torpor use, winter survival and fecundity
Source: eLife. 2018 Feb 20;7:e31225. doi: 10.7554/eLife.31225 (PMC5819945; doi:10.7554/eLife.31225)
Supplement: Supplementary file 6. — Body mass changes were computed between post-hibernation and the start of early breeding, and between the start of early breeding and the start of late breeding. p-Values shown in bold correspond to statistically significant and interpretable values. [file elife-31225-supp6.docx]

**Table S6.** Parameters of analyses of variance for the effects of time, group and diet on body mass and body mass changes of juvenile garden dormice during the post-hibernation breeding period. Body mass changes were computed between post-hibernation and the start of early breeding, and between the start of early breeding and the start of late breeding. p-values shown in bold correspond to statistically significant and interpretable values.

| Response variable | Term | χ^2^ | p-value |  |
| --- | --- | --- | --- | --- |
|  |  |  |  |  |
| Body mass (g) | Time  Group  Diet  Time x Group  Time x Diet  Group x Diet | 383.26  7.82  0.11  2.38  0.93  2.39 | **< 0.001**  **< 0.01**  0.74  0.30  0.63  0.12 |  |
|  |  |  |  |  |
| Body mass change (g) | Time  Group  Diet  Time x Group  Time x Diet  Group x Diet | 2.27  0.22  0.93  3.28  0.28  3.62 | 0.13  0.64  0.33  0.08  0.60  0.06 |  |
|  |  |  |  |  |
|  |  |  |  |  |
