# Supplementary material for: Implications of being born late in the active season for growth, fattening, torpor use, winter survival and fecundity
Source: eLife. 2018 Feb 20;7:e31225. doi: 10.7554/eLife.31225 (PMC5819945; doi:10.7554/eLife.31225)
Supplement: Supplementary file 7. — Groups differing significantly (p<0.05, Tukey’s post-hoc comparisons) are denoted by different superscripts. [file elife-31225-supp7.docx]

**Table S7.** Means and standard deviations of body mass at post-hibernation, start of early breeding and start of late breeding, as well as body mass changes between pre-hibernation and the start of early breeding, and between the start of early breeding and the start of late breeding of the experimental animal groups, according to the time of birth (early-born ‘EB’, late-born ‘LB’) and feeding treatment (*ad libitum ‘*AL’ and intermittently fasted ‘IF’). Groups differing significantly (p<0.05, Tukey’s post-hoc comparisons) are denoted by different superscripts.

| Variable | Time | Group | Mean ± SD |
| --- | --- | --- | --- |
|  |  |  |  |
| Body mass (g) | Post-hibernation | EB-AL  EB-IF  LB-AL  LB-IF | 65.7 ± 0.4^ab^  63.7 ± 1.0^ab^  59.3 ± 0.6^a^  60.5 ± 1.7^ab^ |
|  | Start early breeding | EB-AL  EB-IF  LB-AL  LB-IF | 79.4 ± 3.1^cd^  74.8 ± 8.1^bcd^  68.5 ± 5.9^ab^  71.3 ± 7.3^bc^ |
|  | Start late breeding | EB-AL  EB-IF  LB-AL  LB-IF | 89.2 ± 7.5^d^  83.2 ± 4.3^cd^  79.4 ± 6.8^bcd^  83.1 ± 3.3^cd^ |
| Body mass change (g) | Post-hibernation  🡪 Start early breeding | EB-AL  EB-IF  LB-AL  LB-IF | 13.7 ± 3.4^a^  11.1 ± 5.8^a^  10.8 ± 2.7^a^  11.6 ± 2.7^a^ |
|  |  |  |  |
|  | Start early breeding  🡪 Start late breeding | EB-AL  EB-IF  LB-AL  LB-IF | 11.6 ± 2.9^a^  4.4 ± 4.7^a^  10.6 ± 4.2^a^  12.7 ± 5.6^a^ |
|  |  |  |  |
|  |  |  |  |
|  |  |  |  |
