# Supplementary material for: Implications of being born late in the active season for growth, fattening, torpor use, winter survival and fecundity
Source: eLife. 2018 Feb 20;7:e31225. doi: 10.7554/eLife.31225 (PMC5819945; doi:10.7554/eLife.31225)
Supplement: Supplementary file 8. — p-Values shown in bold correspond to statistically significant and interpretable values. [file elife-31225-supp8.docx]

**Table S8.** Parameters of generalized linear models (in the case of breeding proportion) or linear models (in the case of litter size and litter mass) for the effects of timing of reproduction, group and diet on the female breeding proportion, litter size and litter mass. p-values shown in bold correspond to statistically significant and interpretable values.

| Response variables | Predictor variables | Estimate  ± SD | Statistical value | p-value |
| --- | --- | --- | --- | --- |
|  |  |  |  |  |
| Breeding proportion | Timing of reproduction  Group  Diet | -20.37 ± 8.83  19.84 ± 9.31  -18.97 ± 9.64 | -2.32  2.14  -1.98 | **< 0.05**  **< 0.05**  **< 0.05** |
|  |  |  |  |  |
| Litter size | Timing of reproduction  Group  Diet | 0.22 ± 0.43  0.03 ± 0.52  -0.07 ± 0.52 | 0.53  0.06  -0.13 | 0.62  0.95  0.90 |
|  |  |  |  |  |
| Litter mass | Timing of reproduction  Group  Diet | 0.13 ± 0.95  -1.10 ± 1.45  -0.89 ± 1.44 | 0.14  -0.76  -0.62 | 0.89  0.46  0.54 |
|  |  |  |  |  |
