# Supplementary material for: Implications of being born late in the active season for growth, fattening, torpor use, winter survival and fecundity
Source: eLife. 2018 Feb 20;7:e31225. doi: 10.7554/eLife.31225 (PMC5819945; doi:10.7554/eLife.31225)
Supplement: Transparent reporting form [file elife-31225-transrepform.docx]

***eLife’s* transparent reporting form**

We encourage authors to provide detailed information *within their submission* to facilitate the interpretation and replication of experiments. Authors can upload supporting documentation to indicate the use of appropriate reporting guidelines for health-related research (see [EQUATOR Network](http://www.equator-network.org/%20)), life science research (see the [BioSharing Information Resource](https://biosharing.org/)), or the [ARRIVE guidelines](http://www.plosbiology.org/article/info:doi/10.1371/journal.pbio.1000412) for reporting work involving animal research. Where applicable, authors should refer to any relevant reporting standards documents in this form.

If you have any questions, please consult our Journal Policies and/or contact us: [editorial@elifesciences.org](mailto:editorial@elifesciences.org).

**Sample-size estimation**

- You should state whether an appropriate sample size was computed when the study was being designed
- You should state the statistical method of sample size computation and any required assumptions
- If no explicit power analysis was used, you should describe how you decided what sample (replicate) size (number) to use

Please outline where this information can be found within the submission (e.g., sections or figure legends), or explain why this information doesn’t apply to your submission:

An appropriate sample size was computed during the study design. We performed a power analysis (R package ‘pwr’) by using published or available data. The main variables of this study were the rate of body growth and changes in body composition, but the power analysis was also applied to other variables such as torpor parameters.

**Replicates**

- You should report how often each experiment was performed
- You should include a definition of biological versus technical replication
- The data obtained should be provided and sufficient information should be provided to indicate the number of independent biological and/or technical replicates
- If you encountered any outliers, you should describe how these were handled
- Criteria for exclusion/inclusion of data should be clearly stated
- High-throughput sequence data should be uploaded before submission, with a private link for reviewers provided (these are available from both GEO and ArrayExpress)

Please outline where this information can be found within the submission (e.g., sections or figure legends), or explain why this information doesn’t apply to your submission:

Given the seasonal constraints and long-term aspect, each experiment was performed once. Hence, biological replications (those related to the animal’s responses) do not really apply to our experimental model. However, technical replicates concerned mainly the measurement of structural growth, i.e. body length, which were assessed three times per individuals for a given time-point. Outliers for body length measurements were excluded based on a maximum difference (> 5 mm) between measurements. This information is mentioned on page 6, lines 31-35. Also, the first 24h of respirometry data were excluded for all animals, since they were considered as habituation time (information stated on page 8, lines 34-35).

**Statistical reporting**

- Statistical analysis methods should be described and justified
- Raw data should be presented in figures whenever informative to do so (typically when N per group is less than 10)
- For each experiment, you should identify the statistical tests used, exact values of N, definitions of center, methods of multiple test correction, and dispersion and precision measures (e.g., mean, median, SD, SEM, confidence intervals; and, for the major substantive results, a measure of effect size (e.g., Pearson's r, Cohen's d)
- Report exact p-values wherever possible alongside the summary statistics and 95% confidence intervals. These should be reported for all key questions and not only when the p-value is less than 0.05.

Please outline where this information can be found within the submission (e.g., sections or figure legends), or explain why this information doesn’t apply to your submission:

For the data statistical analyses, we used linear models (for body mass, body length and energetic variables) and generalized linear mixed models for specific diet effect on torpor and activity times. We employed linear mixed-effects models to analyze effects of group, diet and time on metabolic rate variables, as well as on body composition before and after hibernation. Finally, generalized linear models were used to test effects of the timing of breeding, group and diet on the breeding probability of the individuals. This information can be found on pages 11-12-13, section ‘Statistical analyses’ of the submission. Raw data are presented in Figures 1-to-5 and in Supplementary figures S1-to-S3. Statistical tests, exact sample sizes and other information are mentioned in figure legends.

(For large datasets, or papers with a very large number of statistical tests, you may upload a single table file with tests, Ns, etc., with reference to sections in the manuscript.)

**Group allocation**

- Indicate how samples were allocated into experimental groups (in the case of clinical studies, please specify allocation to treatment method); if randomization was used, please also state if restricted randomization was applied
- Indicate if masking was used during group allocation, data collection and/or data analysis

Please outline where this information can be found within the submission (e.g., sections or figure legends), or explain why this information doesn’t apply to your submission:

For each time of birth (early-born and late-born), animals were assigned randomly to experimental groups (i.e. ad-libitum or intermittently fasted), with the restriction that body mass of the two animals’ groups matches each other at the start of the experiments. Masking was not used during neither group allocation, nor data collection and analysis. This information can be found on page 5, section ‘Protocol overview’ of the submission.

**Additional data files (“source data”)**

- We encourage you to upload relevant additional data files, such as numerical data that are represented as a graph in a figure, or as a summary table
- Where provided, these should be in the most useful format, and they can be uploaded as “Source data” files linked to a main figure or table
- Include model definition files including the full list of parameters used
- Include code used for data analysis (e.g., R, MatLab)
- Avoid stating that data files are “available upon request”

Please indicate the figures or tables for which source data files have been provided:

No additional data files were added.
